# Walk the Talk: The Unfinished and Urgent Task of Revising Top‐Line Family Planning Indicators, 30 Years After ICPD

**DOI:** 10.1111/sifp.70007

**Published:** 2025-04-23

**Authors:** Jamaica Corker, Michelle Weinberger, Aisha N.Z. Dasgupta, Elizabeth A. Sully

## Abstract

The 1994 International Conference on Population and Development transformed the family planning (FP) field. Yet, three decades later, global FP monitoring remains anchored in the same core indicators: contraceptive use, unmet need, and demand satisfied. Despite decades of well‐established critique, these measures have seen little substantive revision. As the global community looks to 2030 and beyond, a coordinated effort is urgently needed to develop a revised set of easily interpretable, top‐line FP indicators. Leveraging recent momentum around advances in FP and contraceptive measurement, this effort must be intentional and focused—ensuring that new indicators better reflect the values and priorities of the field—and should be guided by principles of simplicity, validity, directionality, and universal applicability. Without action, the FP community risks continued reliance on outdated measures or the adoption of impractical, unvalidated indicators, weakening future accountability for rights‐based FP. We call on key stakeholders—FP advocates, donors, and global organizations—to prioritize and invest in the development and validation of robust new top‐line FP indicators over the next three years, to ensure their integration into post‐2030 global agenda setting.

## INTRODUCTION

Although the International Conference on Population and Development (ICPD) transformed the family planning (FP) field, global monitoring still relies on the same top‐line contraceptive indicators that predate the 1994 Cairo conference. These indicators—contraceptive prevalence, unmet need, and, later, demand for family planning satisfied—have remained largely unchanged since their development in the 1960s’ Knowledge, Attitudes, and Practice surveys. Despite widespread critiques going back more than 30 years, they continue to serve as the primary headline FP monitoring metrics. It is time for the FP field to move beyond these long‐recognized limitations and build on ongoing efforts to develop and validate a new suite of global FP indicators.

We urge the FP community to seize the opportunity presented at the 30th anniversary of ICPD to prioritize the unglamorous but critical task of redefining FP's persistent top‐line indicators to ensure they better reflect and meet the needs of the field. A coordinated and concerted effort is needed immediately to land on a revised suite of headline FP indicators in time for integration into the post‐2030 agenda setting. We must ensure that the FP field does not continue to rely on these same measures for future global goal setting and monitoring simply because it is not prepared to propose viable alternative indicators.

In this commentary, we call on the FP field, an integral part of sexual and reproductive health and rights (SRHR), to commit to the deliberate and onerous process of establishing a new suite of top‐line indicators within three years. Failing to do so risks codifying, yet again, outdated FP indicators that do not reflect the ICPD Program of Action or its future direction in the post‐2030 global agenda. On the other hand, proposing aspirational indicators that are not ready for use in global monitoring could compromise future accountability for rights‐based family planning. We argue that, over the next few years, the FP field must prioritize reaching a consensus on a set of indicators for the next generation of global monitoring in the post‐2030 era. This process must be deliberate, disciplined, and inclusive of diverse global perspectives. We propose that the development of new FP global monitoring indicators be guided by four key principles: simplicity, validity, directionality, and universal applicability.

## THE IMPERFECT MOMENTUM OF FAMILY PLANNING'S TOP‐LINE INDICATORS

The 1994 ICPD broadened the FP field to include reproductive choice and rights, explicitly calling out the harm caused by contraceptive targets, and fundamentally changed the narrative of FP. While ICPD laid out a bold and transformative new vision of sexual and reproductive health, including for FP, there have not been substantive changes over the past 30 years in how FP progress is measured at the highest levels of international goal setting, with the field continuing to rely on contraceptive prevalence, unmet need for family planning, and demand for family planning satisfied^1^ (Campbell‐White et al. 2006; Senderowicz 2020).

There has been some evolution in reporting methodologies and slight adjustments to FP global monitoring frameworks since ICPD. Initially, the Millennium Development Goals (MDGs) did not include any family planning indicators, but in 2005—more than a decade after ICPD—MDG 5B was expanded to include contraceptive prevalence rate (CPR) and unmet need. The Sustainable Development Goals (SDG) later introduced “demand for family planning satisfied by modern methods” as a key aggregate indicator, but this simply combines contraceptive prevalence and unmet need—representing a shift in reporting, not measurement. Furthermore, while there are recommended benchmarks for the SDG demand satisfied indicator, there is no clearly defined target (Choi et al. 2015). Additionally, while SDG 3.7.1 aims to “ensure universal access to sexual and reproductive health care services, including for family planning” by 2030, the demand satisfied indicator does not directly capture this goal. Family Planning 2020 (FP2020) and Family Planning 2030 (FP2030) introduced a broader measurement framework with a more comprehensive suite of core indicators to track progress but still maintained a primary focus on additional users of modern methods, modern CPR (mCPR), unmet need, and demand satisfied. Over this same period, the methodology for measuring these indicators has seen some evolution, shifting from reporting values based on the most recent household survey to statistical models for estimates and projections (Alkema et al. 2013). Despite these tweaks and minor adjustments to measurement methodologies and reporting frameworks—such as the inclusion of unmarried women or an expanded range of contraceptive methods—core global FP indicators remain largely unchanged, continuing to focus on the same three indicators: contraceptive use, unmet need, and demand satisfied.

At the same time, the shortcomings of these three indicators have been well‐documented for decades (Fabic 2022, Speizer et al. 2022, Rominski and Stephenson 2019; Bradley and Casterline 2014; Pritchett 1996; Dixon‐Mueller and Germain 1992). Although the ICPD established a framework for rights‐based family planning, it has long been recognized that the FP indicators we rely on are often problematic, prone to misinterpretation, and, in some cases, concealing or even contributing to rights‐based violations. Contraceptive prevalence is informative for understanding the overall levels of contraceptive use in a population and has important links to other health and development outcomes. However, it can be misconstrued, or even harmful, when it is set as a singular goal indicator or assumed that increases in contraceptive use alone represent better outcomes.

Unmet need has long faced both conceptual and practical criticism. Conceptually, it reflects a survey‐assigned mismatch between stated fertility intentions and contraceptive behaviors, often misinterpreted or misused to indicate demand for or lack of access to contraception. Practically, using unmet need as an outcome misapplies a population‐level proxy for demand to individuals, potentially obscuring (or leading to) rights violations by assuming that all women wanting to avoid pregnancy should necessarily use contraception without directly considering their preferences. For instance, while “zero unmet need” has been used as a rallying call (e.g., Guttmacher's Adding it Up report, UNFPA's goal to “end unmet need for family planning”), framing the elimination of unmet need as a goal implies that all nonusers wanting to avoid pregnancy should adopt contraception, even though some do not express a desire to do so. Moreover, by focusing solely on the population not using a contraceptive method, unmet need also overlooks potential dissatisfaction and unwanted contraceptive use among current users. Demand satisfied simply takes these two indicators, contraceptive use and unmet need—each with its own well‐established shortcomings—and constructs a new measure that is neither based on actual demand nor actual satisfaction of that demand.

Some argue that the durability of these top‐line global FP measures reflects the legacy of population control (Senderowicz 2020). While these indicators can be misconstrued or misused to support a population control agenda, we believe this perspective overemphasizes the role of population control objectives in current FP measurement. Instead, we argue that the persistence of mCPR, unmet need, and demand satisfied in global FP monitoring frameworks largely reflects path dependency. For international initiatives, indicators needed to be selected based on the feasibility of reporting on them across a wide range of countries. At the national level, surveys that report these indicators provide critical data that are consistently collected and easily comparable across time and place.

The continued reliance on these same top‐line indicators also reflects the practical utility of certain indicators both within and outside the FP field. For example, contraceptive prevalence has likely persisted as the key high‐level FP indicator not due to population control objectives but largely because it is easy to understand and serves as a key input for FP program planning, supply forecasting, and budgeting at both country and global levels. Thus, despite the ICPD pivot to rights‐based family planning, these indicators have had strong staying power primarily because they are already collected in existing surveys, have direct relevance for program planning, and are easy to compare. Such a well‐established and convenient path dependency is hard to break.

As a result, the FP field has been left with the same durable, though imperfect, top‐line indicators for nearly half a century, despite the progress made in centering reproductive choice and rights since ICPD. Now more than ever—with the deadline looming for defining the next generation of global development goals by 2030—it is imperative to break that pattern. The FP community must seize the opportunity presented by the setting of the post‐2030 global agenda to tackle the difficult and uncomfortable task of creating and coalescing around new top‐line indicators that will better guide the global FP agenda, regardless of future shifts in the enabling or political environment. The result must be a set of robust and streamlined global indicators that reflect the values and goals of family planning work and will strengthen, rather than compromise, rights‐based family planning in an uncertain future.

## TOWARDS REVISED GLOBAL FP INDICATORS

The shift to the post‐2030 agenda necessitates not only rethinking or reconceptualizing high‐level FP indicators but also proposing alternatives. Recently some notable adjustments have been proposed to the definitions and calculations of contraceptive use, unmet need, and demand satisfied (e.g., Bradley et al. 2012, Kantorová et al. 2020; Moreau et al. 2019; Fabic and Becker 2017). Although these incremental changes are important steps for improving key FP measures, they alone are insufficient for fundamentally shifting the field's measurement to a rights‐based approach at the highest level.

There have also been efforts to create new high‐level contraceptive‐related indicators, such as preference‐aligned fertility management (Holt et al. 2023) and Senderowicz's (2020) contraceptive autonomy indicator. These efforts, among others, are commendable and may hold promise as alternative high‐level measures but require further refinement, validation, and broader socialization before they can be incorporated into current data collection platforms or practically implemented as global indicators. Other existing or adapted measures that are gaining traction, such as intention to use, are unlikely candidates for new global monitoring indicators as they lack clear directionality and continue to prioritize increased contraceptive use as the primary outcome, limiting their relevance for broader rights‐based family planning goals. However, features of these and other measures can—and should—inform the development of the next generation of top‐line FP measures.

Indeed, there have been efforts to coordinate FP measurement improvement and innovation that can provide important inputs to the development of the next generation of FP indicators. For example, members of the FP2030 Performance, Monitoring, and Evaluation Working Group recently issued a call to refine current FP measures and develop new ones that incorporate perspectives from women and their partners (Speizer et al. 2022). They offer a set of recommendations for this process, identify promising measures, and issue a call to action to the broader FP field to develop and test new FP metrics. They do not, however, propose new indicators or a timeline. Likewise, the current IUSSP scientific panel “Rethinking Family Planning Measurement with a Reproductive Justice Lens” aims to inform and support the development roadmap for reconsidering and revising FP measurement but does not aim to propose new measures. Similarly, the recently launched Family Planning Measurement Advancement Convening Series (FP MACS) aimed to promote and coordinate efforts to advance FP measurement, but with a focus on different levels and areas beyond revisions to the top‐line global monitoring indicators (MOMENTUM 2024a). Despite these multiple efforts underway for promoting and coordinating FP measurement innovation, there remains a critical gap in the proposal, development, and validation of new top‐line measures to meet the next generation of FP global monitoring needs.

Our call builds on the current momentum for and work to date around rethinking FP measures, including ongoing efforts focusing on global monitoring frameworks, but takes it one step further. We urge the global FP community to leverage the impending post‐2030 development agenda deadline to prioritize and allocate resources over the next three years to the process of defining and agreeing on a new suite of top‐line FP indicators. To achieve this, the field must be prepared in the immediate future to come together and make deliberate, and often difficult, decisions on which limited set of indicators to rely and report on at the highest level.

New indicators should be straightforward, valid and easily measurable, and universally relevant for tracking progress toward rights‐based FP as both an imperative and a broader health and development goal. The process of establishing a new suite of top‐line FP indicators should thus be guided by the principles of simplicity, validity, directionality, and universal applicability:

- *Simplicity* requires balancing the complexity of FP with the need for easily measurable indicators that can be understood both within and outside of the FP community. It also calls for a pragmatic approach to using or collecting data that can draw from existing sources or easily be integrated with large‐scale data efforts.
- *Validity* means top‐line measures must reflect their intended concepts and avoid “problematic language and misuse of common FP measures” (Speizer et al. 2022). It is essential for the FP field to clearly define and measure what we value, ensuring that our measures and indicator titles reflect those values. Top‐line measures must be defined in a way such that changes over time can be clearly interpreted as progress toward a goal. This requires moving beyond merely renaming or refining current measures of unmet need or demand satisfied.
- *Directionality* ensures that top‐line indicators provide meaningful and actionable information, with a clear interpretation of the direction of change to measure and benchmark progress. While a single indicator will never be able to capture all the complexities of FP, measures must provide a general sense of the directionality of progress. Directionality is also critical to future‐proof key indicators, as the lack of clear definitions of success can ultimately undermine progress by allowing countries to ignore vaguely defined goals or claim achievements without meaningful change.
- Finally, high‐level measures must have *universal applicability* to be relevant in all settings, not only in the low‐ and middle‐income developing countries (LMICs) that are the focus of international FP work and partnerships. Rights‐based and justice‐informed FP measures are universally relevant and challenges to meeting them are not limited to developing countries. Global FP indicators should be well‐placed for adoption by global monitoring frameworks (e.g., UN, SDGs) and applicable for reporting across all countries, and as relevant in Uganda as in the United States of America.

No set of high‐level indicators can encompass all the nuances and complexities inherent in family planning—or any field. Instead, streamlined, easily interpretable top‐line measures must be complemented by robust local FP measurement ecosystems and context‐specific indicators. These should include person‐centered measures of preference, choice, and agency, along with indicators of the enabling environment and access to accurate, reliable information.

Moreover, top‐line indicators alone cannot provide a complete picture of FP progress, and we must be realistic about which indicators can feasibly and effectively monitor progress toward rights‐based FP. For example, while many in the FP field advocate moving away from CPR, this may be met with resistance from country stakeholders who rely on the indicator for planning purposes. If this is the case, a top‐line indicator that incorporates CPR—but does not report it in isolation—may be a viable compromise. Similarly, while integrating new indicators into Health Management Information System (HMIS) may be ideal, routine health systems data are better suited for tracking service use than for measuring complex demand and need dynamics, which require surveys. Ultimately, the FP field must strike a balance between capturing complexity and ensuring simplicity to create global measures that safeguard rights‐based family planning while remaining actionable and easily understood.

## NOW OR NEVER MIND (UNTIL AT LEAST 2045)

The ICPD's 30th anniversary and the upcoming formation of the post‐2030 global development agenda provide a key moment for FP indicator reform. The FP field must agree on and propose new top‐line measures by 2028 (if not before), in time to be integrated into the next round of global goal setting, or risk having the same top‐line indicators from decades ago accompany us over the next decade and into ICPD+40 discussions. Prioritizing this is especially critical in an era of shrinking resources and increasingly unfavorable political environments for FP, SRHR, and global development, lest we lose momentum now and find ourselves in 2030 with the same outdated indicators that fail to reflect the evolving needs of the field, setting us back rather than moving us forward.

A concerted, coordinated effort must begin now to meet this timeline. The refinement, validation, and socialization needed to reach a broad consensus on robust top‐line FP indicators will be a time‐consuming process, particularly if integrating new indicators into existing surveys or HMIS requires changes to data requirements. If this is left to the last minute, the field runs the risk of proposing aspirational but impractical indicators that will make monitoring progress difficult. If new indicators necessitate modifications to existing data sources, these must be ready by 2030; otherwise, the field will be delayed in its ability to track the very indicators it sets forth. The defunding of the Demographic and Health Surveys (DHS) and significantly reduced support for the Performance Monitoring for Action (PMA) project leaves FP without its key sources of global monitoring data and suggests that new and independent sources of FP data may be necessary moving forward. This presents a monumental challenge—but also a new opportunity—for the field to recalibrate its FP data collection approach in tandem with the development of new FP indicators.

While financial support for the process to refine FP's top‐line global monitoring indicators is essential, it need not be substantial, as much of the groundwork is already in place. Even under the reality of diminished resources for FP and measurement work amid the shifting donor landscape, limited funding could deliver significant returns. Despite ongoing funding cuts and a precarious future funding outlook, continuing to advance the next generation of headline FP indicators remains crucial to ensuring robust and future‐proofed metrics. Leadership for this effort could come from a major global institution, such as United Nation's Population Fund (UNFPA), WHO, or FP2030, or be coordinated by a temporary committee established for this purpose.

If the FP field does not come together now to propose a new set of FP key indicators, we will very likely fall back yet again on the well‐established and familiar measures that have persisted in global goal setting, locking in contraceptive prevalence and unmet needs as the field's global guiding indicators for another generation. Should this happen, the global FP community will have no one to blame but itself. We urge the FP field not to make perfection the enemy of progress and to start now to make the hard choices and compromises necessary for meaningful change in top‐line global contraceptive measurement for this next round of global goal setting.

## CALL TO ACTION

We call on the FP field to prioritize, over the next three years, the development of a revised suite of no more than three top‐line FP measures for global monitoring and goal setting. This process of creating new top‐line indicators may not be the most inspiring or innovative work in FP measurement, particularly in comparison to recent impressive programmatic and demand‐side measurement innovations (e.g., Rothschild et al. 2021; Moreau et al. 2020; Choi et al. 2016), but it is essential to ensuring meaningful progress in global monitoring and goal setting. This effort will require a comprehensive but disciplined approach, focusing on selecting the most relevant high‐level indicators, rather than proposing a multitude of complicated new indicators across all levels of FP monitoring.

The process of redefining FP's limited set of global headline indicators can—and should—happen side‐by‐side with ongoing work to reconceptualize FP measures for different levels of policy, programming, and reporting. Several key initiatives are already underway to develop new FP measures, including global convenings focused on new FP measures (IUSSP 2024) and sexual and reproductive agency (UNFPA 2024). These types of convenings are critical for advancing measurement change at all levels of FP programs and policy, but they must be paired with a focused effort to develop concrete top‐line FP indicators that align with global monitoring needs. Without this deliberate, complementary approach, broader measurement innovations may not translate into meaningful change at the highest level.

Developing these new top‐lin FP indicators cannot be solely an academic exercise or privilege discussions and decisions by Western researchers and organizations. The process must be inclusive, incorporating diverse participation from a broad range of stakeholders globally. Country‐level voices are essential in this undertaking to provide informed, practical perspectives to ensure indicators are practical, inform national priority setting, can integrate into HMIS and local surveys, and meet countries’ monitoring needs. Existing work to enhance country engagement, including recommendations from the FP MACS June 2024 meeting on centering locally driven FP measurement priorities (MOMENTUM 2024b), can provide guidance in this regard. Efforts to make FP indicators more universally applicable and relevant, however, must extend country‐level engagement beyond the usual focus on LMICs or donor‐recipient countries to incorporate diverse perspectives from practitioners and programmers worldwide.

The development of a new set of global FP indicators also requires funders, intergovernmental organizations, and governments to recognize the urgency of this initiative and allocate resources specifically toward the development and validation of new top‐line FP indicators. While much of the foundational work around rethinking and proposing new FP measures has been done, a coordinated final push is now essential to deliver a robust set of FP indicators in time for the post‐2030 global agenda‐setting process. To that end, we call on major FP donors and influential organizations to prioritize funding over the next few years to meet critical milestones needed to complete this effort in time to integrate new indicators into the next round of global goal setting. The first step is to define the mandate and identify the lead organization. A key milestone in this effort will be the International Conference on Family Planning planned for November 2025, which provides a crucial platform to convene global and local FP measurement stakeholders, establish a clear mandate, and outline concrete steps toward finalizing new top‐line indicators.

The right set of top‐line indicators is imperative to keep FP relevant on the global agenda and hold countries and international programs accountable to SRHR goals. The window of opportunity to develop and propose new top‐line FP measures for the post‐2030 agenda, and to move the field away from its headline indicator path dependency and closer to the ICPD principles, is fast closing. The FP field must seize this opportunity, as we look back at ICPD and beyond 2030, to ensure we are prepared to guide and drive the measurement change needed to support rights‐based FP and meaningful progress toward universal access to FP worldwide.

## Note

1 Contraceptive prevalence is the percentage of women of reproductive age who are using (or whose partner is using) a method of contraception. Unmet need for family planning is the percentage of women of reproductive age who want to avoid pregnancy for the next two years or more but are not using contraception. Demand for family planning satisfied is the percentage of women of reproductive age who want to avoid pregnancy who are currently using or whose partner is currently using a method of contraception. These indicators can be reported for all contraceptive methods (modern, traditional, and folkloric) or specific to modern methods (e.g., demand for family planning satisfied with modern methods).
